# Characterization of lamina propria remodeling in pediatric eosinophilic esophagitis using second harmonic generation microscopy

**DOI:** 10.1186/s41231-024-00170-2

**Published:** 2024-03-22

**Authors:** Ezekiel J. Haugen, Andrea K. Locke, Hernán Correa, Justin S. Baba, Anita Mahadevan-Jansen, Girish Hiremath

**Affiliations:** 1 Vanderbilt Biophotonics Center, Vanderbilt University, Nashville, TN 37232, USA; 2 Department of Biomedical Engineering, Vanderbilt University, Nashville, TN 37232, USA; 3 Department of Chemistry, Vanderbilt University, Nashville, TN 37232, USA; 4 Division of Pediatric Pathology, Monroe Carell Jr. Children’s Hospital at Vanderbilt, Vanderbilt University Medical Center, Nashville, TN 27232, USA; 5 Division of Pediatric Gastroenterology, Hepatology and Nutrition, Monroe Carell Jr. Children’s Hospital at Vanderbilt, Vanderbilt University Medical Center, Nashville, TN 37232, USA

**Keywords:** Eosinophilic esophagitis, Fibrosis, Second Harmonic Generation Microscopy, Multiphoton Imaging, Extracellular matrix, Histopathology

## Abstract

Eosinophilic esophagitis (EoE) is a chronic inflammatory condition characterized by an intense infiltration of eosinophils into the esophageal epithelium. When not adequately controlled, eosinophilic inflammation can lead to changes in components of the extracellular matrix (ECM) of the lamina propria. Particularly, alterations to the collagen fiber matrix can lead to lamina propria fibrosis (LPF), which plays an important role in the fibrostenotic complications of EoE. Current approaches to assess LPF in EoE are prone to inter-observer inconsistencies and provide limited insight into the structural remodeling of the ECM. An objective approach to quantify LPF can eliminate inter-observer inconsistencies and provide novel insights into the fibrotic transformation of the lamina propria in EoE. Second harmonic generation (SHG) microscopy is a powerful modality for objectively quantifying disease associated alterations in ECM collagen structure that is finding increasing use for clinical research. We used SHG with morphometric analysis (SHG-MA) to characterize lamina propria collagen fibers and ECM porosity in esophageal biopsies collected from children with active EoE (*n* = 11), inactive EoE (*n* = 11), and non-EoE (*n* = 11). The collagen fiber width quantified by SHG-MA correlated positively with peak eosinophil count (*r* = 0.65, *p* < 0.005) and histopathologist scoring of LPF (*r* = 0.52, *p* < 0.005) in the esophageal biopsies. Patients with active EoE had a significant enlargement of ECM pores compared to inactive EoE and non-EoE (*p* < 0.005), with the mean pore area correlating positively with EoE activity (*r* = 0.76, *p* < 0.005) and LPF severity (*r* = 0.65, *p* < 0.005). These results indicate that SHG-MA can be utilized to objectively characterize and provide novel insights into lamina propria ECM structural remodeling in children with EoE, which could aid in monitoring disease progression.

## Background

Eosinophilic esophagitis (EoE) is an allergen-mediated chronic inflammatory condition of the esophagus estimated to affect 1 in 1500 children in the United States [1]. If left undiagnosed or ineffectively managed, EoE can lead to fibrostenotic complications such as dysphagia, esophageal narrowing, and esophageal food impaction that may require urgent endoscopic intervention [2–5]. An improved understanding of fibrostenosis in EoE and the development of objective methods to detect early fibrotic changes are needed to improve clinical outcomes.

The diagnostic hallmark of EoE is an intense influx of eosinophils into the esophageal epithelium, defined as ≥ 15 eosinophils/high-power field (eos/hpf). Prolonged eosinophilic inflammation can lead to lamina propria fibrosis (LPF), which is regarded as one of the key changes associated with fibrostenotic EoE and is a result of excessive deposition of extracellular matrix (ECM) components such as collagen (i.e., type I collagen) with increased fibroblast activation and proliferation [2, 6–8]. Currently, the assessment of LPF in biopsies collected from EoE patients relies on manual and subjective interpretation of lamina propria (LP) remodeling (e.g. increased collagen fiber size or deposition) by pathologists and researchers using traditional staining methods such as hematoxylin and eosin (H&E), trichrome and picrosirius red stains [6, 9, 10]. Regrettably, these approaches lack quantitative measurements of ECM remodeling that occurs with LPF and are prone to inter-observer inconsistencies, which limits the understanding of LPF pathobiology and can yield inconsistent diagnosis [11, 12]. As such, an unbiased approach is needed to better characterize the mechanisms of fibrostenosis in EoE and improve the clinical assessment of lamina propria health.

Second harmonic generation (SHG) microscopy is a label-free imaging technique that allows for accurate, unbiased, and automated characterization of fibrillar collagen in tissues. Second harmonic generation occurs when two photons simultaneously interact with a molecule and produce a photon of exactly twice the energy. Type I and II collagen provide significant SHG due to their highly non-centrosymmetric triple-helix structure and fibrillar alignment [13, 14]. Exploiting this effect, SHG microscopy is used for the optical characterization of collagen at submicron scales in many physiologic conditions and pathologic states [14, 15]. Morphometric analysis of SHG images can then be used to quantify alterations in the geometry and porosity of the collagen fiber matrix [16]. These properties can provide novel insights into collagen fibrogenesis, cross-linking, and degradation; cell migration and proliferation; and ECM biomechanical properties [16–19]. For the esophagus, SHG microscopy has been employed to characterize alterations in subepithelial collagen content and morphology in Barrett’s esophagus, squamous cell carcinoma and adenocarcinoma [20–22]. However, SHG microscopy has not been used to investigate LPF in EoE.

We hypothesize that SHG is an accurate and objective method to quantify structural changes in the esophageal lamina propria of EoE patients. As such, in this study, SHG microscopy with morphometric analysis (SHG-MA) was employed to examine the relationship between markers of structural remodeling in the lamina propria and EoE activity status. Additionally, we investigated the association between SHG-MA parameters and conventional histopathology scoring of EoE-based tissue changes assessed by a pathologist. Lamina propria collagen fiber width, fiber SHG/TPEF (two-photon excitation fluorescence) ratio, and extracellular matrix porosity were found to correlate with EoE diagnosis, the severity of eosinophilic inflammation, and LPF as assessed by an independent pathologist (gold standard). These findings provide new insights into lamina propria ECM remodeling in EoE and indicate that SHG-MA has potential to be a valuable tool for accurately characterizing LPF to aid in monitoring disease progression.

## Methods

### Study subjects and clinical assessment

Esophageal biopsies with adequate lamina propria, as determined by an expert pathologist (H.C.), were analyzed from 33 children (≤ 18 years) who had undergone esophagogastroduodenoscopy (EGD) with biopsies at Monroe Carell Jr. Children’s Hospital at Vanderbilt University Medical Center for their clinical care between 2017 and 2019. During EGD, esophageal mucosal biopsies (3–4 each from the proximal and distal esophagus) were obtained using conventional biopsy forceps (Endo-Jaw, Olympus Medical Systems Corp, Tokyo, Japan) and submitted for H&E staining per the institutional protocol.

The clinical H&E sections of the biopsies were further analyzed as approved by the Vanderbilt University Medical Center Institutional Review Board (IRB #202459). The sections were assessed by an expert pathologist (H.C.) for peak eosinophil count (PEC) per high-power-field (hpf = 0.237mm^2^) and for the grade (or the severity) of the involvement of esophageal tissue per the validated EoE Histology Scoring System (EoEHSS) [9]. The EoEHSS assesses eight EoE-relevant histologic features: eosinophilic inflammation (EI), basal zone hyperplasia (BZH), eosinophilic abscess (EA), eosinophilic surface layering (ESL), dilated intercellular spaces (DIS), surface epithelial alteration (SEA), dyskeratotic epithelial cells (DEC), and LPF. Each feature was scored on a 4-point scale (0–3), with 0 representing normal features and 3 denoting the most severe or extensive pathology. The mean score of the number of features assessed yields the total EoEHSS score.

The study groups were defined per the 2011 Consensus statement [23]. Children with active EoE (aEoE) had one or more symptoms of esophageal dysfunction and a PEC of ≥ 15 eos/hpf in at least one of the multiple esophageal biopsies acquired, after excluding other causes of esophageal eosinophilia. Those with a previous diagnosis of EoE and a PEC < 15 were classified as having inactive EoE (iEoE). The non-EoE control group (controls) consisted of children without a prior diagnosis of EoE but with symptoms of esophageal dysfunction and not meeting the histologic criteria for EoE (PEC < 15).

### Second harmonic generation imaging

**Fig. 1:**
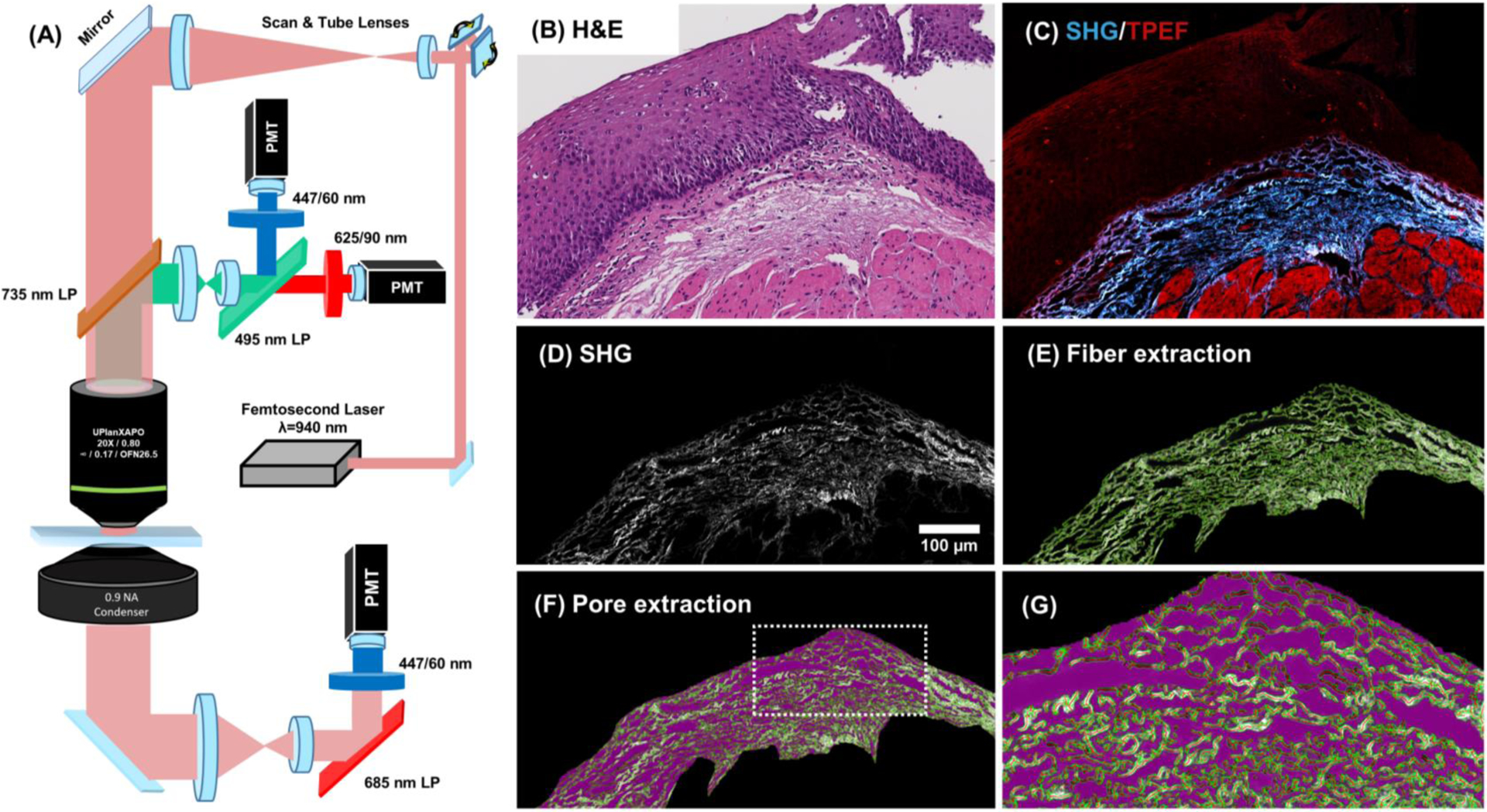
Nonlinear microscopy and morphometric analysis of esophageal lamina propria in pediatric biopsies. **a** A nonlinear microscope is used to scan **b** 5 μm thick H&E sections, providing **c** two-photon excitation fluorescence images (red) and second harmonic generation (SHG) images with collagen fiber contrast (blue). The images are linearly contrast adjusted for visualization (Scale bar: 100 μm). **d** Backward SHG image depicts the collagen fiber distribution in the lamina propria. **e** A ridge detection algorithm (Steger method) is used for automated fiber segmentation. **f** Regions not containing fibers, termed pores (magenta), are extracted using Moore-Neighbor tracing and the white rectangle is magnified in panel **g**

The H&E sections (5 μm thick) from 33 esophageal biopsies were scanned under a multimodal nonlinear microscope previously described [24]. Briefly, a femto-second laser (Insight DS+, Spectra-Physics, USA) tuned to 940 nm was used for excitation, with ~12.5 mW average power on the specimen (Fig. 1a). Images (1024 × 1024 pixels; 381 × 381 μm) were collected via galvanometric scanning and a 20X objective (Olympus UPLXAPO, 20 × 0.8 NA). The images were stitched using MosaicJ in FIJI/ImageJ [25, 26] across the entire lamina propria and used for quantitative analysis. A blue filter (447/60 nm | Semrock, USA) was used to isolate SHG emission (470 nm) in the forward and backward directions to visualize collagen. A red filter (625/90 nm | Semrock, USA) was used to collect two-photon excitation fluorescence (TPEF), which allowed for visualization of the epithelium and underlying muscularis mucosa (if present). The signals were collected using photomultiplier tubes (Amplified PMT, Thorlabs, USA) with 5–10 μs pixel dwell times, with the intensities of each image being normalized to its dwell time. A digital H&E image and its corresponding SHG and TPEF stitched images from a patient biopsy section are shown in Fig. 1b and c, respectively.

### Quantitative image analysis

Stitched images from each sample were batch processed in MATLAB (Natwick, MA, USA) and ImageJ. First, the images were segmented such that only the LP was present for further analysis. A 1-pixel radius Gaussian blur was applied to each image to reduce shot noise. Morphometric features in the lamina propria, including collagen fiber width and ECM porosity, were then quantified. The SHG images were converted to 8-bits so that the gray values were within the accepted range for the ridge detection algorithm in Fiji/ImageJ [27, 28] (Fig. 1d). The ridge detection algorithm was then applied with the linewidth set to 6 pixels (~2.23 μm) [27, 29] (Fig. 1e). The output lists of identified fibers, with their respective widths, were used to calculate each patient sample’s mean LP fiber width (μm). The fibers detected from the ridge detection algorithm and a median threshold of the SHG signal within the LP were combined to generate a binary mask of collagen fibers. Through visual inspection, this method was found to robustly separate collagen fibers from pores (regions absent of collagen). The percentage of lamina propria taken up by collagen fibers termed the fiber coverage, was then calculated for each specimen. Additionally, the SHG/TPEF ratio and the forward-to-backward (F/B) SHG ratio were calculated for pixels within the binary mask of fibers. For porosity quantification, the fiber mask was inverted to provide a binary image of the pores (Fig. 1g). Each pore was then identified using Moore-Neighbor tracing [30]. The list of pores (with their respective areas) for each patient specimen was used to calculate the mean pore area (μm^2^). The number of pores, along with the total area of the lamina propria, was used to calculate pore density (# pores / mm^2^).

### Statistical analysis

To determine if the fiber and porosity metrics were distinct between patients with aEoE, iEoE, and controls, analysis of variance (ANOVA) was used with a multiple comparison test using Tukey’s criterion [31]. A Spearman correlation was then used to quantify the relationships between the fiber and porosity metrics from SHG-MA and conventional histopathology metrics (including PEC, BZH, LPF, and total EoEHSS grade). Lastly, to determine the diagnostic accuracy of detecting EoE using the most significant SHG-MA metric we employed receiver operating characteristic (ROC) curve analysis.

## Results

### Cohort characteristics

Overall, 22 children had EoE (aEoE = 11 and iEoE = 11), and 11 were controls. The cohort’s median (interquartile range) age was 8 (4–12) years. Expectedly, PEC was significantly different in aEoE compared to iEoE and non-EoE controls (Table 1). Similarly, aEoE had higher LPF scores compared to iEoE and controls. The EoEHSS grade, indicating the overall severity of the esophageal tissue involvement, was also higher for aEoE compared to iEoE and controls (Table 1).

### Correlating SHG-MA fiber and porosity features with EoE diagnosis

**Fig. 2:**
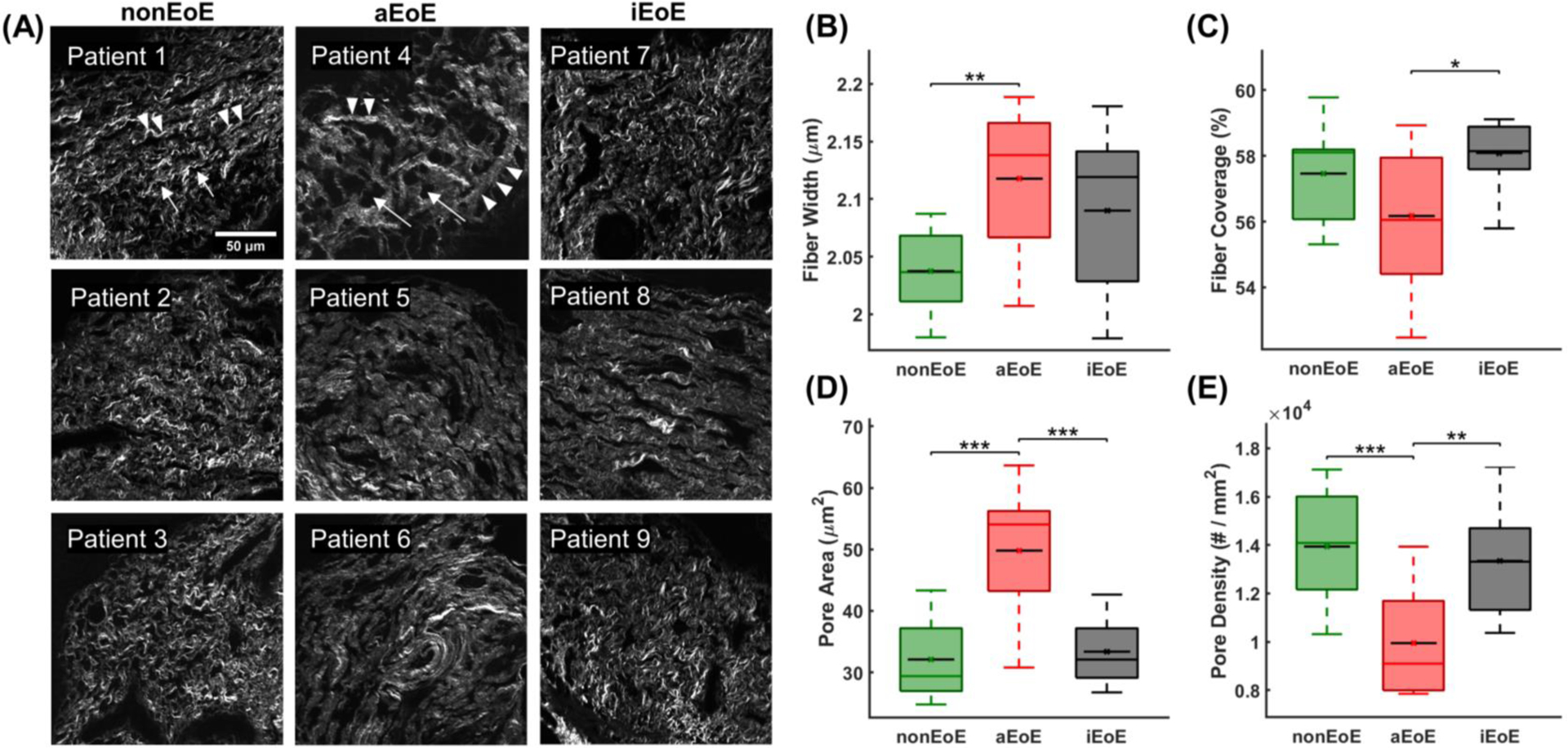
Dependence of esophageal lamina propria morphology on eosinophilic esophagitis (EoE) status. **a** Mean normalized second harmonic generation images of esophageal lamina propria from nine patients depict morphological differences in the collagen fiber matrix that are dependent on EoE activity status (Scale bar: 50 μm). Arrows point to pores and arrow heads point to fibers. **b** Esophageal lamina propria fiber width (μm) extracted using a ridge detection algorithm is significantly higher in active EoE (aEoE) compared to non-EoE controls. **c** Fiber coverage (%) is significantly higher in inactive EoE (iEoE) compared to aEoE. **d** Lamina propria pore area (μm^2^) and **e** pore density (# pores / mm^2^) are significantly different in aEoE compared to iEoE and non-EoE controls. Box plot displays median line across the box and mean line in black with a center mark, while the top and bottom of the box represent the 25th and 75th percentiles. The whiskers represent the farthest data points not considered an outlier (1.5× the respective interquartile range). (* *p* < 0.05, ** *p* < 0.01, *** *p* < 0.005)

**Fig. 3:**
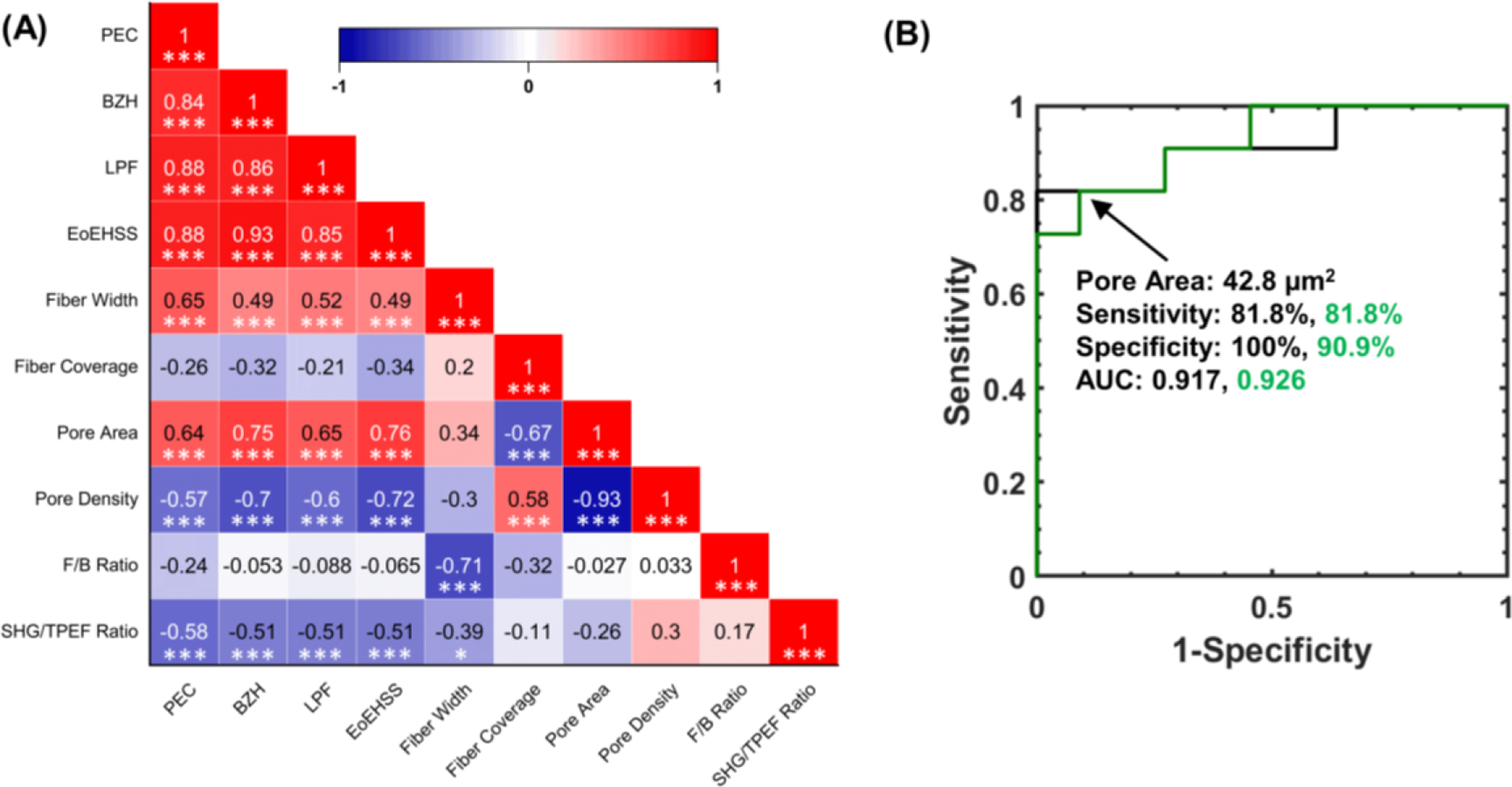
**a** Correlation between second harmonic generation image parameters and eosinophilic esophagitis features assessed by a pathologist. Coefficient values are given by a Spearman’s rank correlation (* *p* < 0.05, ** *p* < 0.01, *** *p* < 0.005). **b** Receiver operating characteristic (ROC) curve analysis to determine the diagnostic accuracy of the mean pore area in differentiating active EoE from inactive EoE samples (black line) and active EoE from nonEoE control samples (green line)

Second harmonic generation images of the lamina propria revealed altered morphology that was dependent on EoE status (Fig. 2a). The lamina propria collagen fiber width was significantly greater in aEoE compared to controls [(mean ± standard deviation): 2.12 ± 0.06 μm vs. 2.04 ± 0.04 μm; *p* = 0.006] (Fig. 2b) but not iEoE (2.09 ± 0.07 μm; *p* = 0.5). The fiber coverage was lower for aEoE compared to iEoE (56.2 ± 2.1% vs. 58.1 ± 1.0%; *p* = 0.02) but was not significantly different from controls (57.5 ± 1.5%; *p* = 0.6) (Fig. 2c). The pore area was significantly higher for aEoE compared to iEoE (50 ± 10 μm^2^ vs. 33 ± 5 μm^2^; *p* < 0.005) and controls (32 ± 6 μm^2^; *p* < 0.005) (Fig. 2d). Lastly, lower pore density was observed in aEoE compared to iEoE (9950 ± 2200 pores/mm^2^ vs. 13,300 ± 2300 pores/mm^2^; *p* < 0.005) and controls (13,900 ± 2400 pores/mm^2^; *p* < 0.005) (Fig. 2e). Ultimately, the most significant SHG-MA metric for detecting aEoE samples was the pore area. Using ROC analysis the pore area was found to differentiate aEoE from iEoE samples with 81.8% sensitivity and 100% specificity and aEoE from nonEoE controls with 81.8% sensitivity and 90.9% specificity, using a threshold of 42.8 μm^2^ (Fig. 3b).

### Correlating SHG-MA features and histopathologist scoring

The lamina propria collagen fiber and porosity metrics strongly correlated with the gold standard histopathology scores (Fig. 3a). The lamina propria collagen fiber width had a significant positive relationship with PEC (*r* = 0.65, *p* < 0.005), BZH (*r* = 0.49, *p* < 0.005), LPF (*r* = 0.52, *p* < 0.005) and total EoEHSS (*r* = 0.49, *p* < 0.005). Likewise, the mean pore area had a significant positive correlation with PEC (*r* = 0.64, *p* < 0.005), BZH (*r* = 0.75, *p* < 0.005), LPF (*r* = 0.65, *p* < 0.005) and total EoEHSS (*r* = 0.76, *p* < 0.005). Conversely, pore density had a significant negative correlation with PEC (*r* = −0.57, *p* < 0.005), BZH (*r* = −0.7, *p* < 0.005), LPF (*r* = −0.6, *p* < 0.005), and total EoEHSS (*r* = −0.72, *p* < 0.005). The F/B ratio of the collagen fibers did not correlate with histopathology scoring but had a strong negative correlation with the fiber width (*r* = −0.71, *p* < 0.005). Lastly, the SHG/TPEF ratio of the collagen fibers had a significant negative correlation with PEC, BZH, LPF, and total EoEHSS (*p* < 0.005).

## Discussion

Understanding the health of esophageal lamina propria has important clinical, diagnostic, and prognostic implications in EoE. Current approaches to characterize the esophageal lamina propria are largely based on visual cues that include increased fiber thickness and density in stained sections [6, 9, 10]. Unfortunately, these approaches are prone to inconsistencies as they are subjective and rely on the training and experience of the pathologist [11]. We present the application of SHG-MA to objectively quantify changes in the lamina propria and observed that the properties of ECM collagen fibers and porosity strongly correlated with EoE activity as determined by an expert pathologist. Specifically, the fiber width and SHG/TPEF ratio, and the pore area and density most significantly correlated with EoE activity status, PEC, BZH, LPF, and total EoEHSS grade. These findings provide novel insights into the structural changes of collagen fibers in esophageal lamina propria affected by EoE. Additionally, they support the application of SHG-MA as an unbiased and automated approach to quantify markers of LPF in EoE.

The collagen fiber width, quantified using SHG-MA, correlated positively with EoE activity and the gold standard histopathology assessment of LPF, which is based on a visual evaluation of the ECM fiber width. The SHG images indicated that the growing fiber width was due to the merging of normal smaller collagen fibers into larger bundles in aEoE (Fig. 2a). This observation aligns with studies that have noted an increase in collagen cross-linking enzymes in aEoE patients [32]. Morphometric analysis revealed that the fiber width and the pore area significantly increased with EoE activity (Fig. 3a), which has been seen in subepithelial tissues undergoing natural (e.g. pregnant cervix remodeling) or pathological (e.g., colon cancer) collagen remodeling characterized by cross-linking [16, 19]. Excessive collagen deposition, often mentioned when describing LPF in EoE [7], was not visually evident in the SHG images. In fact, the quantified percentage of lamina propria taken up by collagen (fiber coverage) had a slight but non-significant negative correlation with EoE severity (Fig. 3a). However, this analysis does not consider total LP volume or thickness, due to the inherent limitation of biopsy sampling, which would be needed to assess total volumetric collagen deposition. Moreover, this result does not preclude an increase in overall ECM fibrous content [33] but instead raises the possibility of the presence of other non-SHG-producing ECM fibers, as indicated by the decreasing fiber SHG/TPEF ratio with EoE. Overall, the results demonstrate that SHG-MA quantification of fiber width alone has significant correlation with the conventional method of manually assessing esophageal LPF in children with EoE.

Novel to our study was the identification of altered ECM porosity during LP remodeling in EoE. Specifically, the mean pore area increased, and the pore density decreased with EoE activity and returned to normal values with inactive EoE. We postulate that the increased pore space, devoid of type I collagen, may be filled with cells, fluid, blood vessels, and other biomolecules that do not produce SHG signals (elastin, soluble collagen, non-fibrillar collagen, etc.). For example, the known increase in eosinophils and lymphoid follicles within the lamina propria of EoE samples [34] may contribute towards the formation of larger pores. Likewise, enhanced cell migration in EoE (i.e., leukocyte and myofibroblast trafficking) may contribute towards increased ECM pore size, which could be further studied by developing bioengineered tissues within the range of quantified fiber and porosity values [7, 17, 35, 36]. However, additional assays will be necessary to identify the most significant driver of altered lamina propria ECM porosity and how it relates to epithelial profibrotic markers (such as transforming growth factor β, α-smooth muscle actin, and periostin) in children with EoE.

Along with the new insights into LP remodeling observed in this study, the significant correlation between fiber and porosity metrics with gold standard histopathology and diagnosis has potential clinical implications. This is evidenced by the significantly greater pore area and lower pore density in aEoE compared to iEoE and control patients (Fig. 2d, e), leading to high diagnostic accuracy in detecting aEoE (Fig, 3b). These findings suggest that the quantitative features extracted from SHG-MA could aid physicians and provide improved intra-observer agreement on LPF progression and treatment efficacy, which could improve patient outcomes. Likewise, SHG-MA could provide objective metrics for relating microscopic changes of the LP to macroscopic changes in esophageal distensibility, as assessed by the endoluminal functional lumen imaging probe [2, 37].

Overall, the results from this study highlight that SHG-MA can provide objective metrics that significantly correlate to gold standard EoE and LPF severity assessments. However, future studies are needed to address the limitations of this work. Methodologically, images were collected from fixed biopsy sections, which does not overcome the clinical limitations of conventional tissue processing and sectioning artifacts, including improper section orientation. As such, future work will focus on scanning fresh bulk biopsies, which is an important advantage of SHG microscopy that can address this pitfall. Another limitation, related to the ex vivo SHG-MA approach is that about 50% of the esophageal biopsies collected using conventional biopsy forceps from pediatric EoE patients have inadequate lamina propria [38]. As such, this method may not be applicable in certain ex vivo situations. Despite these weaknesses, our study has several strengths. This is the first study to apply SHG-MA to objectively quantify the fibrotic transformation of lamina propria in patients with EoE. This approach can complement existing methods to better understand the pathobiology of lamina propria fibrosis and remodeling in EoE. Future studies will look to combine SHG with other multiphoton modalities (e.g. third harmonic generation [39] and stimulated Raman microscopy [40]) to quantify changes in the lamina propria, beyond collagen, toward improved sensitivity to EoE and LPF. Ultimately, the results from this study lay the foundation for the development of an in vivo endoscopic nonlinear imaging probe for the assessment of the health of esophageal lamina propria in children with EoE. This in vivo approach has the potential to overcome the limitation associated with inadequate sampling of the esophageal lamina propria using conventional biopsy forceps and could in the future serve as a modality for real-time assessment of the esophageal lamina propria in EoE.

## Conclusions

Using SHG-MA, we objectively characterized and quantified esophageal lamina propria remodeling in children with and without EoE. This study adds to our understanding of esophageal LPF in EoE and demonstrates that SHG-MA holds promise to serve as an accurate, unbiased, and automated approach to quantify markers of LPF. Ongoing studies aim to investigate how the SHG-MA metrics correlate with epithelial profibrotic markers, esophageal distensibility assessed by the endoluminal functional lumen imaging probe, and EoE therapies. In the future, the use of SHG-MA for in vivo endoscopic detection of LPF in EoE will be explored.

## Figures and Tables

**Table 1:** Peak eosinophil count (PEC) and histopathology scores for esophageal biopsies.

	Cohort (*n* = 33)			*P* ^a^	
	nonEoE (*n* = 11)	aEoE (*n* = 11)	iEoE (*n* = 11)	aEoE vs. nonEoE	aEoE vs. iEoE
**PEC (eos/hpf)**	0 (0–0)	62 (44–116)	3 (0–4)	<0.005	<0.005
**Eosinophilic Inflammation**	0 (0–1)	2 (2–3)	1 (0–1)	<0.005	<0.005
**Basal Zone Hyperplasia**	0 (0–1)	3 (3–3)	1 (0–1)	<0.005	<0.005
**Eosinophilic Abscess**	0 (0–0)	1 (0–3)	0 (0–0)	<0.005	<0.005
**Eosinophil Surface Layering**	0 (0–0)	1 (0–3)	0 (0–0)	<0.005	<0.005
**Dilated Intercellular Spaces**	0 (0–0)	3 (2–3)	0 (0–1)	<0.005	<0.005
**Surface Epithelial Alteration**	0 (0–0)	0 (0–2)	0 (0–0)	0.02	0.03
**Dyskeratotic Epithelial Cells**	0 (0–0)	0 (0–1)	0 (0–0)	0.02	0.02
**Lamina Propria Fibrosis**	0 (0–0)	2 (2–3)	0 (0–1)	<0.005	<0.005
**EoEHSS Grade**	0 (0–0.13)	1.63 (1.13–2.25)	0.13 (0.13–0.50)	<0.005	<0.005

Results given as median (interquartile range).

a Analysis of Variance with Tukey multiple comparison tests.

*aEoE* Active EoE, i*EoE* Inactive EoE

## Data Availability

All data collected during the study are available from the corresponding author upon reasonable request.

## Abbreviations

EoE: Eosinophilic esophagitis
LPF: Lamina propria fibrosis
ECM: Extracellular matrix
H&E: Hematoxylin and Eosin
SHG: Second harmonic generation
SHG-MA: Second harmonic generation with morphometric analysis
TPEF: Two photon excitation fluorescence
EGD: Esophagogastroduodenoscopy
IRB: Institutional review board
PEC: Peak eosinophil count
hpf: high-powered field
eos: eosinophils
EI: eosinophilic inflammation
BZH: basal zone hyperplasia
EA: eosinophilic abscess
ESL: eosinophilic surface layering
DIS: dilated intercellular spaces
SEA: surface epithelial alteration
DEC: dyskeratotic epithelial cells
aEoE: active eosinophilic esophagitis
iEoE: inactive eosinophilic esophagitis
F/B: forward-to-backward
ANOVA: analysis of variance
EoEHSS: eosinophilic esophagitis histology scoring system
